# Multi-Modal Electrophysiological Source Imaging With Attention Neural Networks Based on Deep Fusion of EEG and MEG

**DOI:** 10.1109/TNSRE.2024.3424669

**Published:** 2024-07-11

**Authors:** Meng Jiao, Shihao Yang, Xiaochen Xian, Neel Fotedar, Feng Liu

**Affiliations:** Department of Systems and Enterprises, Stevens Institute of Technology, Hoboken, NJ 07030 USA.; Department of Systems and Enterprises, Stevens Institute of Technology, Hoboken, NJ 07030 USA.; H. Milton Stewart School of Industrial and Systems Engineering, Georgia Institute of Technology, Atlanta, GA 30332 USA.; Epilepsy Center, Neurological Institute, University Hospitals Cleveland Medical Center, Cleveland, OH 44106 USA; Department of Neurology, Case Western Reserve University School of Medicine, Cleveland, OH 44106 USA.; Department of Systems and Enterprises and the Semcer Center for Healthcare Innovation, Stevens Institute of Technology, Hoboken, NJ 07030 USA

**Keywords:** Brain source localization, EEG/MEG source imaging, multi-modal deep learning, deep attention network, information fusion

## Abstract

The process of reconstructing underlying cortical and subcortical electrical activities from Electroencephalography (EEG) or Magnetoencephalography (MEG) recordings is called Electrophysiological Source Imaging (ESI). Given the complementarity between EEG and MEG in measuring radial and tangential cortical sources, combined EEG/MEG is considered beneficial in improving the reconstruction performance of ESI algorithms. Traditional algorithms mainly emphasize incorporating predesigned neurophysiological priors to solve the ESI problem. Deep learning frameworks aim to directly learn the mapping from scalp EEG/MEG measurements to the underlying brain source activities in a data-driven manner, demonstrating superior performance compared to traditional methods. However, most of the existing deep learning approaches for the ESI problem are performed on a single modality of EEG or MEG, meaning the complementarity of these two modalities has not been fully utilized. How to fuse the EEG and MEG in a more principled manner under the deep learning paradigm remains a challenging question. This study develops a Multi-Modal Deep Fusion (MMDF) framework using Attention Neural Networks (ANN) to fully leverage the complementary information between EEG and MEG for solving the ESI inverse problem, which is termed as *MMDF-ANN*. Specifically, our proposed brain source imaging approach consists of four phases, including feature extraction, weight generation, deep feature fusion, and source mapping. Our experimental results on both synthetic dataset and real dataset demonstrated that using a fusion of EEG and MEG can significantly improve the source localization accuracy compared to using a single-modality of EEG or MEG. Compared to the benchmark algorithms, MMDF-ANN demonstrated good stability when reconstructing sources with extended activation areas and situations of EEG/MEG measurements with a low signal-to-noise ratio.

## I. Introduction

Electrophysiological source imaging (ESI), also known as EEG/MEG source localization, is a non-invasive neuroimaging technique that aims to determine specific brain sources responsible for generating electrophysiological signals that can be detected by EEG/MEG sensors from the scalp [1]. Compared with other non-invasive neuroimaging techniques, such as functional near-infrared spectroscopy (fNIRS), functional magnetic resonance imaging (fMRI), computed tomography (CT), and positron emission tomography (PET), EEG and MEG have excellent temporal resolution up to milliseconds. EEG also enjoys the advantages of easy portability and low cost. Furthermore, EEG/MEG provides direct measurements of neuronal activities, while fMRI measures blood oxygen level dependent (BOLD) signals [2], which are secondary measurements of metabolic signals. Owing to these advantages, ESI enables researchers and clinicians to uncover latent brain signals characterizing rapid neuronal dynamic activities, which serves as a fundamental tool for neuroscience studies and clinical diagnosis [1], [3], [4], [5], [6], [7], [8]. However, the ill-posed nature of the ESI inverse problem makes it challenging to uniquely determine the active underlying brain sources. Additionally, the recorded EEG/MEG signals are inevitably contaminated by measurement noises, which complicates the accurate reconstruction of source distributions. During the past decades, numerous algorithms have been proposed to seek a unique solution to the ESI inverse problem (see the comprehensive and insightful review paper by Dr. Bin He [1] and references therein).

A traditional approach is to constrain the solution space by utilizing prior information about the configurations of the source signal [9], [10], [11], [12]. Based on this assumption, the minimum norm estimate (MNE) [13] was introduced by imposing the $\ell_{2}$ norm as a regularization term to promote a solution with minimum energy while explaining the EEG/MEG measurements. MNE exhibits excellent performance in the localization of superficial or near-surface sources, but when applied to deep sources, MNE may face challenges. To deal with this problem, several variants and extensions of MNE have been developed, including weighted MNE (wMNE) [13], standardized low resolution electromagnetic tomography (sLORETA) [14] and dynamic statistical parametric mapping (dSPM) [15], etc. These variants were designed to enhance the original MNE by incorporating additional information, such as the depth sensitivity and sensor noise covariance. The methods guided by $\ell_{2}$-norm tend to provide spatially diffuse source estimates. To encourage the sparseness of source solutions, the $\ell_{1}$ norm was proposed, termed as the minimum current estimate (MCE) [16], which promotes sparse source solutions. Bore et al. proposed to apply the $\ell_{p}$ norm where p<1 on the source signal and the $\ell_{1}$ norm on the data fitting error term [17] to obtain sparse sources inferred from EEG with artifact contamination. Babadi et al. developed a greedy pursuit algorithm to iteratively solve the sparse MEG source localization [18]. Gramfort et al. presented a combination use of the $\ell_{2}$ prior and the $\ell_{p}$ norm where p≤1, termed as the mixed norm estimate (MxNE), to encourage an adaptive balance between sparsity and smoothness of the source activation [9]. Yang et al. used low-frequency components of spatial graph Fourier filters to help classical ESI methods to estimate extended brain sources [19]. Another widely used approach for brain source localization is beamformer [20], which is an adaptive spatial filter that can be used to localize neural activities originating within a specific location while attenuating activities originating from other locations by adjusting the weights of the filter [21]. One of the most commonly used beamforming techniques is the linearly constrained minimum variance (LCMV) beamformer [22]. For instance, Hong et al. presented an LCMV beamformer cooperated with a source suppression strategy for localization of coherent sources [23]. Samadzadehaghdam et al. proposed a SParse LCMV (SP-LCMV) by introducing $\ell_{1}$ norm regularization of the beamformer output in the cost function to encourage the reconstruction of sparse sources [24]. Moreover, to encourage the localization of multiple sources, Moiseev et al. derived source localizers based on multiple constrained minimum variance (MCMV) filters [22]. Herdman et al. proposed an MCMV-based multi-step iterative approach, called MIA, to reconstruct source activity for simulated and real EEG data [25]. More implementations of LCMV and MCMV beamformers can be found in [22] and [26].

Recently, as deep learning approaches have gained increasing popularity, various deep learning frameworks have been investigated to solve the ESI inverse problem in a data-driven way. A key advantage of deep learning approaches is they can directly learn the mapping from the scalp EEG/MEG measurements to the underlying brain sources, eliminating the necessity for pre-specifying the regularization terms. Additionally, once a deep learning model is well-trained, it allows online reconstruction of source signals with EEG/MEG recordings as input in a highly accurate and efficient way. Most of the proposed deep learning frameworks to solve ESI are based on an end-to-end architecture, which leverages convolutional neural networks (CNNs) or Long Short-Term Memory (LSTM) units. For instance, Hecker et al. [27] designed a deep architecture called ConvDip, leveraging 2D CNN layers for precise localization of a varying number of sources. Craley et al. [28] introduced SZTrack, utilizing a 1D CNN encoder alongside bidirectional LSTM (BiLSTM) units for automatic tracking of epileptic seizure activity and localization of seizure zones. Sun et al. [29] proposed DeepSIF by employing residual blocks and LSTM units to perform spatiotemporal estimation of underlying source dynamics. Jiao et al. proposed a graph Fourier transform based Bi-LSTM framework for electrophysiological source imaging [30]. Huang et al. [31] presented a data-synthesized spatiotemporally convolutional encoder-decoder network (DST-CedNet) to learn a robust mapping from EEG/MEG measurements to sources. Furthermore, Liang et al. [32] developed a novel framework called SI-SBLNN for solving the ESI inverse problem by incorporating sparse Bayesian learning with deep neural network.

Existing publications indicate that deep learning frameworks have significant superiority in improving the accuracy of source localization. However, most deep learning models employed a single modality of EEG or MEG to solve the ESI inverse problem, and few studies have leveraged a multimodal integration of simultaneously recorded EEG and MEG, which can benefit the accuracy of ESI given the complementary measurements of the two modalities. Fusion of MEG and EEG reconstruction results in the decision level is usually suboptimal, and how to design an early fusion framework in the context of deep learning paradigm remains a challenging problem. In this study, improved upon our previous work with new attention mechanism [33], we propose a new Multi-Modal Deep Fusion framework using Attention Neural Network, termed as *MMDF-ANN*, where EEG and MEG signals are integrated through early fusion with a specially designed deep learning architecture. Our main contributions are highlighted as follows:

- We proposed a new MMDF-ANN framework for brain source imaging, which employed two CNN modules to separately extract complementary information from EEG and MEG.
- The unique design of EEG and MEG input matrices largely preserves the spatial locations of EEG and MEG sensors, which has been shown to be very effective for solving the ESI problem. Besides, the dilated convolution filter was introduced to expand the receptive field of CNN without adding layers, allowing for more effective capture of spatial dependencies.
- We designed a channel-wise attention module to generate proper weights for feature maps, enabling the adaptive integration of complementary information from two neural signal modalities of EEG and MEG.
- Comprehensive experiments have been conducted on simulated data and real data, showing the superior performance of the proposed MMDF-ANN against the state-of-the-art methods, particularly in the case of extended sources and situations when EEG/MEG measurements are contaminated with a high level of noises.

## II. Problem Statement and Related Work

In this section, we first introduce the forward and inverse problem of ESI in Section II-A, then we highlight two paradigms of ESI algorithms in Section II-B and Section II-C which motivated our method.

### A. Problem Statement

In order to estimate the brain source activation patterns based on the scalp EEG/MEG measurements, an EEG/MEG forward model needs to be established in advance to characterize the mapping from the source space to the EEG/MEG sensors. Solving the “ESI forward problem” produces a *leadfield* matrix, in which each element reflects how the electrophysiological signals generated by neural activities in each brain region influence the EEG/MEG signals measured by each sensor. This relationship can be described by a linear equation:

(1) Y=LS+ε,

where $Y\in R^{C\times K}$ is the recorded EEG and/or MEG, C is the number of electrodes, K indicates time points, $S\in R^{N\times K}$ is the source signal from N brain regions, $L\in R^{C\times N}$ is the leadfield matrix, and $\varepsilon \in R^{C\times K}$ is the measurement noise.

Solving the “ESI inverse problem” requires calculating S based on the measured Y and the leadfield matrix L by solving Eq. (1). However, the ESI inverse problem is ill-conditioned as the number of electrodes is far less than that of brain regions (i.e., C≪N), resulting in infinite source solutions. In order to deal with the ill-posed problem of ESI, traditional approaches attempt to impose regularization constraints to restrict solutions to a subspace that satisfies specific prior assumptions on the source structure [3], [34]. In this case, S can be obtained by solving Eq. (2):

(2) $$S=\underset{S}{argmin}\frac{1}{2}∥Y-LS{∥}_{F}^{2}+\lambda R\left(S\right),$$

where $∥\cdot {∥}_{F}$ is the Frobenius norm. The first term is called *data fitting* error, which is introduced to find the solution that explains the EEG/MEG measurements. The second term is called *regularization* term, which is imposed to obtain a source solution that is constrained to a prior assumption. For example, MCE encourages a sparse solution by adopting the $\ell_{1}$ norm. MNE and its variants (wMNE, sLORETA, dSPM, etc.) promote a diffuse and smooth source distribution by introducing the $\ell_{2}$ norm. λ is a hyperparameter added to control the balance between data fitting error minimization and regularization.

### B. Extended Source Imaging

When a localized assemble of neurons fires, due to the volume conductivity property of the brain, the neighboring sources are most likely to be activated, generating a spatially smoothed activation pattern, which is called source extents or extended source activation. Previous works leveraged the spatial smoothness configuration of source signals based on 3D triangular meshes to promote the source extent estimation [35], [36], [37], [38], [39]. Ding proposed a Total Variation (TV) matrix V derived from brain 3D meshes as a sparse constraint in the transform domain (first order differential spatial space) to encourage similarity of neighboring source activities, thus enabling reconstruction of source extents [40]. The extended source activation has been integrated into multiple ESI frameworks [11], [41], [42], [43]. Given that the epileptogenic zone (EZ) exhibits a focal activation with spatial continuity, the TV term is particularly useful for the localization of EZs. For instance, Sohrabpour et al. [44] proposed the fast spatiotemporal iteratively reweighted edge sparsity minimization (FAST-IRES) algorithm to estimate the extended sources by balancing the source sparsity and edge sparsity while fitting the scalp EEG measurements, and further validated using MEG [45].

### C. Complementarity Between EEG and MEG

According to previous studies [46], [47], [48], EEG and MEG have been shown to be complementary in sensitivity to cortical source orientations. To be specific, MEG exhibits more sensitivity to tangential sources, while EEG can accurately reflect activities of both radial and tangential components [49]. Besides, EEG exhibits high sensitivity to conductivity uncertainties. The electric signals detected by EEG are typically attenuated and smeared due to the low conductivity of the skull, which may further limit the accuracy of source imaging. By contrast, MEG measures the magnetic field generated by electrical activities in the brain, which is barely contaminated by conductivity changes [50]. Previous studies have proposed several EEG/MEG fusion techniques, for instance, the signal-to-noise ratio (SNR) transformation was utilized in [51] and [52] to convert EEG and MEG data into the common SNR domain. Based on this, Ding and Yuan [53] developed a sparse ESI approach with the integration of EEG and MEG to facilitate the reconstruction of complex brain activities. Henson et al. [54] presented an empirical Bayesian scheme where the source solutions from each modality were fused under the common generative model. Baillet et al. [55] proposed a method for cooperative processing of MEG and EEG by selectively weighting their leadfields to minimize the mutual information between them. Given that the complementary information between the EEG and MEG modalities is under-exploited [33], utilizing simultaneously recorded EEG and MEG provides a potential possibility to improve the accuracy and robustness of ESI algorithms [56].

## III. Method

### A. Proposed Framework

**Fig. 1.**
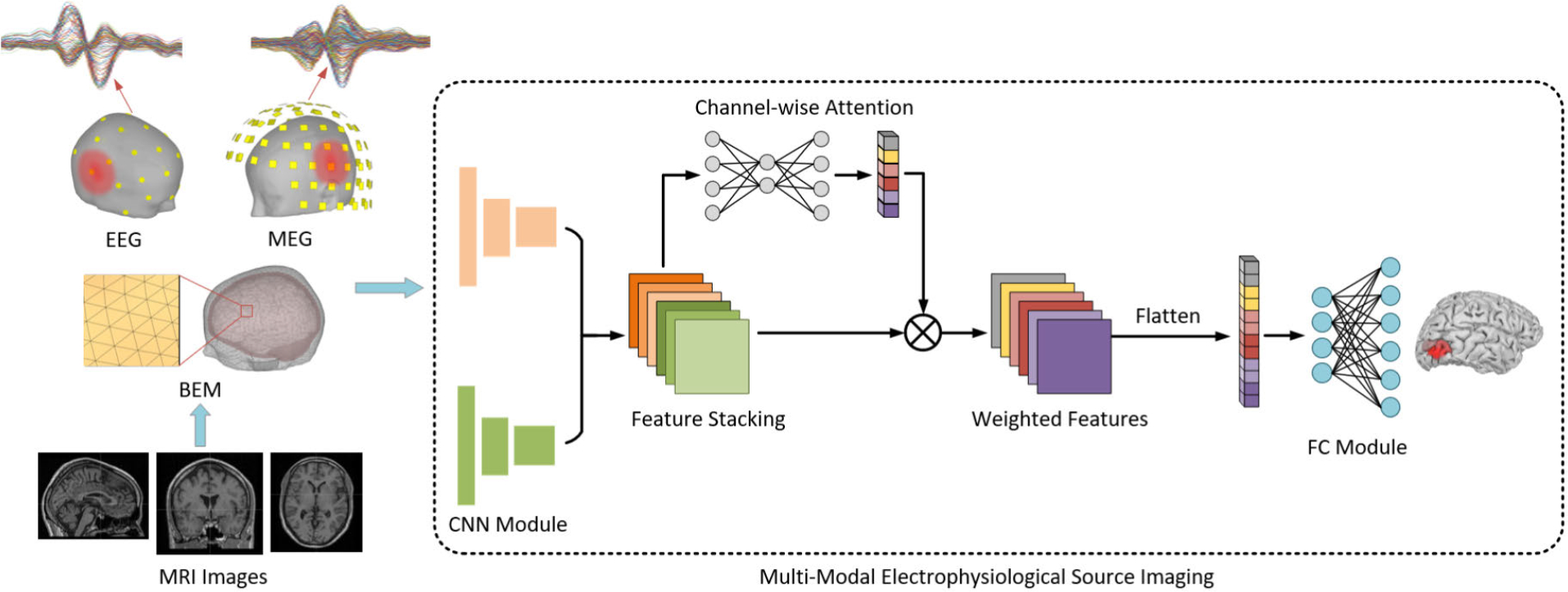
Illustration of the proposed framework: Multi-Modal Deep Fusion framework using Attention Neural Networks (MMDF-ANN).

The MMDF-ANN architecture, as illustrated in Fig. 1, draws inspiration from the input design of ConvDip [27], where the EEG measurements were first interpolated to a matrix according to spatial locations of EEG electrodes, then fed into a 2D convolutional architecture for feature extraction. In this study, both EEG and MEG measurements are separately interpolated to two distinct matrices and further set as inputs of two individual 2D CNN modules. Both CNN modules share identical hyperparameter configurations, employing the dilated convolution filter [57] in which the dilation rate is set to 2 to strategically expand the receptive field without increasing CNN depth. The receptive field is defined as the spatial extent within the input space that contributes to the activation of a neuron in CNN. In the early layers of a CNN, the receptive field is limited and can only capture local features. As the network structure deepens, the receptive field expands, allowing the network to extract more abstract and high-level features by aggregating a broader range of information. Our work employs the dilated convolution, where empty “spaces” are inserted between kernel elements, allowing the kernel to capture information over a more extensive spatial range without the necessity of increasing the CNN depth, thereby mitigating the expansion of model parameters. The dilation rate indicates the extent of kernel expansion, and it is worth noting that when the dilation rate is set to 1, the dilated convolution operation can be regarded as a regular convolution operation.

Given that feature maps derived from distinct CNN channels contribute differently to the source estimation, a self-attention mechanism is introduced to produce proper weights for feature maps [58], [59]. The feature maps $U=\left[u_{1},u_{2},\ldots ,u_{C}\right]$ extracted from C channels are first aggregated to a set of channel descriptors through a global average operation in each feature map, defined as $z_{c}={\bar{u}}_{c}$ for the *c*-th feature map, where ${\bar{u}}_{c}$ is the mean value of the matrix $u_{c}.\;z_{c}$ is the obtained descriptor for the c-th channel of convolution filter. Next, the obtained channel descriptors $z={\left[z_{1},z_{2},\ldots ,z_{C}\right]}^{T}$ are fed into a two-layer fully connected (FC) neural network module to generate the attention weights denoted as w, expressed as $w=\sigma \left(W_{2}\delta \left(W_{1}z\right)\right)$ where $W_{1}\in R^{\frac{C}{r}\times C}$ and $W_{2}\in R^{C\times \frac{C}{r}}$ are two learnable weight matrices, r is the reduction ratio, δ represents the ReLU activation, σ represents the sigmoid activation, $w={\left[w_{1},w_{2},\ldots ,w_{C}\right]}^{T}$ is the obtained weight vector, where the *c*-th element $w_{c}$ represents the generated weight for the feature map $u_{c}$. In MMDF-ANN, the feature maps extracted from different channels were first assigned proper weights learned from the two-layer fully connected neural network, and then the weighted feature maps were flattened to a set of vectors. Next, a concatenation operation was applied to these feature vectors, and finally, the fused feature was fed into a fully connected module with each output representing the activation of each source in the brain. With the designed architecture, the MMDF-ANN framework is able to adaptively leverage the information derived from both EEG and MEG modalities and conduct early fusion in the latent feature space for the ESI inverse problem.

### B. Loss Function

In deep learning, the loss function serves as the objective or goal that the model tries to minimize during training. The design of the loss function reflects in which direction the model is optimized, which can significantly affect the model’s convergence and performance. The first loss function for deep learning models applied to solve the ESI inverse problem is the mean squared error (MSE), which is defined as:

(3) $$L_{mse}=\frac{1}{K}\sum_{i=1}^{K}{\left\|s_{i}-s_{i}^{\prime }\right\|}_{2}^{2},$$

where $s_{i}$ and $s_{i}^{\prime }$ with i∈{1,…,K} respectively represent the ground truth and the estimated source signal from the proposed MMDF-ANN. The MSE loss can effectively quantify the discrepancy between $s_{i}$ and $s_{i}^{\prime }$ by measuring their Euclidean distance, in which the geodesic distance between brain regions is not considered. In other words, the MSE loss imposes the same penalty on two source estimations where one is distributed next to the ground truth location while the other one is further away, resulting in quite different localization errors. To effectively reduce the localization error, we proposed the *topological loss* to assign an appropriate penalty for source estimates based on their geodesic distance, which is defined as:

(4) $$\begin{array}{l} L_{t}=\frac{1}{N\;\times \;N}\sum_{i=1}^{K}\|E⊙M{\|}_{F}^{2},\\ E=\left(s_{i}-s_{i}^{\prime }\right){\left(s_{i}-s_{i}^{\prime }\right)}^{T}, \end{array}$$

where ⊙ represents the element-wise multiplication. $E\in R^{N\times N}$ is a symmetric error matrix, in which the element $e_{ij}$ indicates the product of estimation errors corresponding to region i and region j.M is a symmetric transformation matrix based on the shortest paths between all brain regions, in which the element $m_{ij}$ is defined as:

(5) $$m_{ij}=\left\{\begin{array}{ll} 0 & i=j,\\ tanh\left(\rho \cdot d_{ij}\right) & j\in \Omega_{i},\\ 1 & \text{otherwise,} \end{array}\right.$$

where $d_{ij}$ is the shortest path between region i and region $j,\;\Omega_{i}$ is the set of all the neighboring regions with the shortest path less than a threshold value to region i,ρ is the kernel width in the range of 0 to 1. Thus, the non-zero elements in M exhibit an “inverse-Gaussian” shaped distribution centered on the diagonal. By introducing M, ESI solutions with different spatial distances from the ground truth can be penalized to varying degrees. When the estimated location corresponding to $s^{\prime }$ is closer to that of s, the topological loss ${ℒ}_{t}$ will provide a small value, otherwise a large value. By including the topological loss, the complete loss function can be defined as:

(6) $$ℒ={ℒ}_{mse}+\alpha {ℒ}_{t},$$

where α is a hyperparameter ranging from 0 to 1 used to balance the above two loss components.

## IV. Numerical Experiments

To fully evaluate the performance of the proposed MMDF-ANN framework, we first carried out extensive experiments on synthetic EEG and MEG data with varying activation patterns. Then, we applied the framework to real EEG/MEG recordings from face perception tasks and epileptic seizures for further validation.

### A. Experiments on Synthetic Data

In this section, we first illustrated the process of solving the ESI forward problem, and then we explained the generation procedure of synthetic data with varying activation configurations. Finally, we conducted comprehensive experiments on the obtained datasets.

#### 1) Realistic Head Model:

The forward brain model is derived from MRI scans from the MNE-Python sample dataset [60]. The MEG system consists of 204 gradiometers and 102 magnetometers based on the configuration of the Neuromag Vectorview system, and the EEG data were simultaneously acquired using a 60-channel electrode cap. The original MRI images were obtained with a Siemens 1.5 T Sonata scanner, and the source surfaces were reconstructed using FreeSurfer [61]. Next, a three-layer head model was constructed based on the boundary element method (BEM), and the source space was defined as a grid containing 1984 dipoles distributed across the cortex. All bad channels in EEG and MEG were removed, and only the magnetometers in MEG were involved in the computation process. This configuration results in an EEG leadfield matrix with dimensions of 59 × 1984 and a MEG leadfield matrix with dimensions of 102 × 1984.

#### 2) Synthetic Data Generation:

**Fig. 2.**
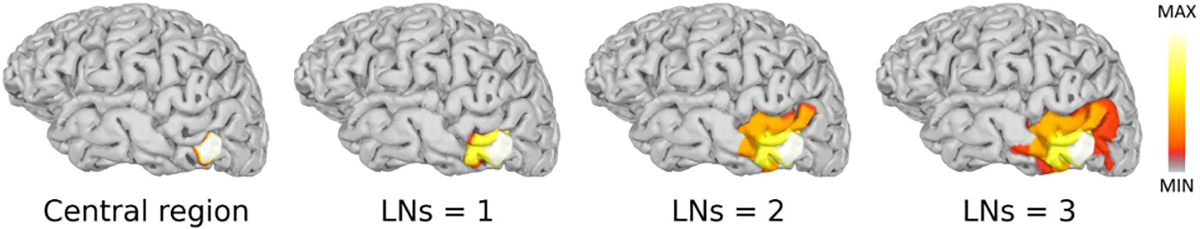
Illustration of activated brain regions based on varying levels of neighborhoods (LNs).

To generate synthetic data, we first generate source signals with different activation patterns. As illustrated in Fig. 2, we introduced three levels of neighborhoods (LNs=1, 2, 3) to indicate different sizes of source extents. Then, we simultaneously activated all brain regions included in the entire “patch”, where the activation intensity of neighboring areas with LNs=1, 2, 3 is respectively configured to be 85%, 70%, and 55% of the central region. After obtaining the source data, the synthetic EEG and MEG data were calculated according to Eq. 1, where the measurement noise was determined based on different SNR levels (SNR = 30 dB, 20 dB, 10 dB). SNR is a measure used to quantify the ratio of the signal power $P_{\text{signal}}$ to the noise power $P_{\text{noise}}$, defined as $SNR=10\;log\left(P_{\text{signal}}/P_{\text{noise}}\right)$.

#### 3) Experimental Settings:

For model training, we first randomly selected 1600 out of 1984 regions to activate under different source configurations. Then all obtained synthetic data sets were employed in the training process of MMDF-ANN, where the designed loss function ℒ in Eq. (6) was utilized with α set to 0.05 and ρ set to 0.005. For model testing, 50 brain regions, distinct from those used for the training set, were randomly selected to form a test set, ensuring that there was no source overlap between the training data and test data. The comparison algorithms include MNE [13], sLORETA [14], dSPM [15], MCMV beamformer [22], ADMM [62], and ConvDip [27]. For MNE, sLORETA, dSPM, MCMV beamformer, and ADMM, the SNR transformation technique [51] was used for EEG/MEG fusion. For ConvDip, since the integration of locations of EEG and MEG sensors was difficult to implement, we first carried out brain source imaging on EEG and MEG independently, and then we averaged the results from both modalities for source reconstruction. The MSE loss function was adopted to guide the model training. All experiments were executed on a Windows PC equipped with an Intel i9 CPU and 64 GB of memory. Training deep learning models employed an NVIDIA V100 GPU with 32 GB of memory.

#### 4) Evaluation Metrics:

The performance of all algorithms was assessed quantitatively using two metrics: *localization error (LE)* and *area under the precision-recall curve (AUPRC)*. LE quantifies the geodesic distance between the region with the maximum amplitude in the reconstructed source area and the center of the actual source patch on cortex meshes using the Dijkstra shortest path algorithm. In this study, the unit of measurement for LE is millimeters (mm). AUPRC assesses the overlap of source extents between the reconstructed and actual sources. Given the sparsity nature of source distributions (unbalanced activated vs non-activated regions), PRC has been suggested to be a more suitable metric than area under the receiver operating characteristics curve (AUROC) for assessing success in imbalanced datasets [63], [64]. Good performance is achieved if LE approaches 0 and AUPRC approaches 1.

#### 5) Source Reconstruction With Single Source:

**Fig. 3.**
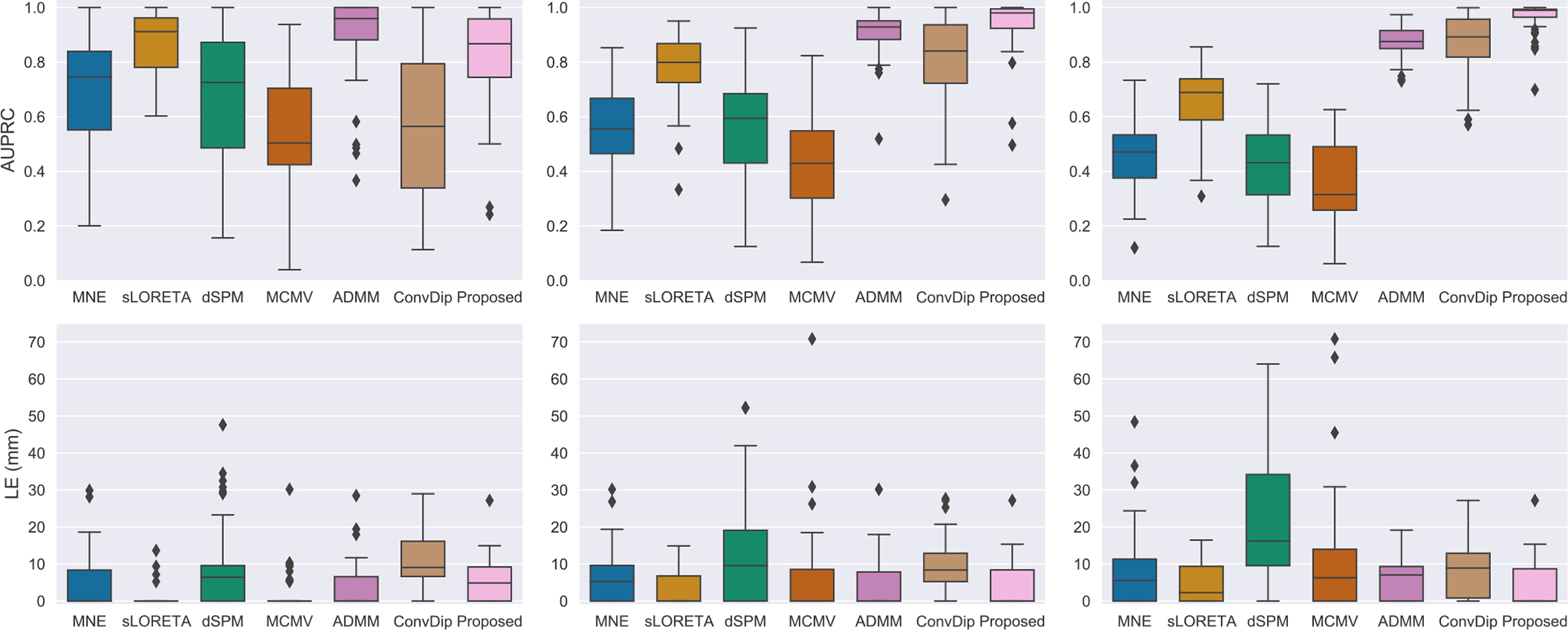
Peformance comparison of different algorithms on AUPRC (on the top) and LE (at the bottom), with SNR of 30 dB for LNs = 1 (on the left), LNs = 2 (in the middle) and LNs = 3 (on the right).

**Fig. 4.**
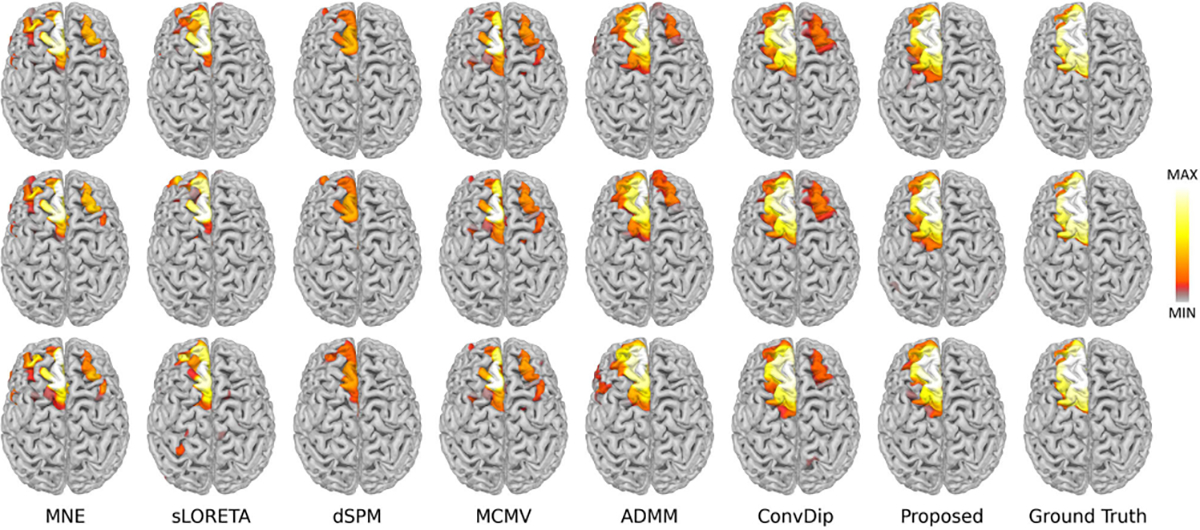
Brain sources reconstruction by different ESI algorithms with 3 levels of neighborhoods for SNR = 30 dB (on the top), SNR = 20 dB (in the middle) and SNR = 10 dB (at the bottom).

The performance comparison between MMDF-ANN and benchmark algorithms on LE and AUPRC is summarized in Table I. The boxplots for different LNs settings with SNR=30 dB are provided in Fig. 3. Reconstructed source distributions for LNs=3 with varying SNR settings (SNR=30 dB, 20 dB, and 10 dB) are shown in Fig. 4. From Table I and Figs. 3–4, we can see that:

1. With the SNR decreases and source range expands, there is a significant increase on LE and a decrease on AUPRC for MNE, sLORETA, dSPM, and MCMV, which means the performance of these algorithms are limited and they are more suitable for localizing sources with concentrated distributions with high SNR EEG/MEG measurements. When an extended area of the source is activated, or the SNR of EEG/MEG data is at a low level, it is difficult for these algorithms to provide reliable solutions. In contrast, ADMM exhibits remarkable stability in LE and achieves superior performance in AUPRC for reconstructing concentrated source extents (LNs=1) across varying SNRs.
2. Moreover, it can be observed in Fig. 3 that with an increase of source extents area (LNs=2, 3), ADMM demonstrates deteriorated performance in AUPRC and LE, whereas ConvDip and the proposed MMDF-ANN both display enhanced performance. This indicates that the *deep learning models are more accurate* than the traditional ones for larger source extent estimation. The reason is that the unique input design enables ConvDip and MMDF-ANN to effectively capture the spatial dependencies present in EEG/MEG measurements and subsequently aggregate the spatial information associated with source activation. What’s more, compared to ConvDip, MMDF-ANN achieves superior performance in all cases, which shows that the designed framework guarantees higher accuracy in source localization.
3. Empirically, it can be seen in Fig. 4 that for source activations distributed on the left frontal, the source estimation provided by ConvDip is partially diffused into the right hemisphere, while the source estimation provided by MMDF-ANN is more concentrated within the left hemisphere. This shows that the proposed loss function can provide better guidance for model training by taking into account the distinct spatial distances between brain regions and penalizing spatial mismatches based on the localization error.

#### 6) Source Reconstruction With Multiple Sources:

**Fig. 5.**
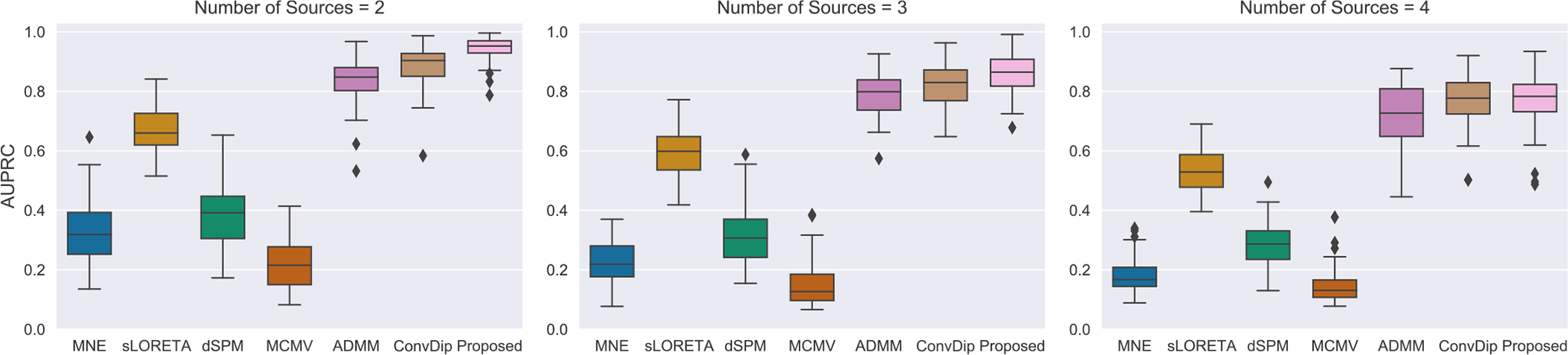
Peformance comparison of different algorithms on AUPRC with SNR = 30 dB and LNs = 3 for multiple sources.

**Fig. 6.**
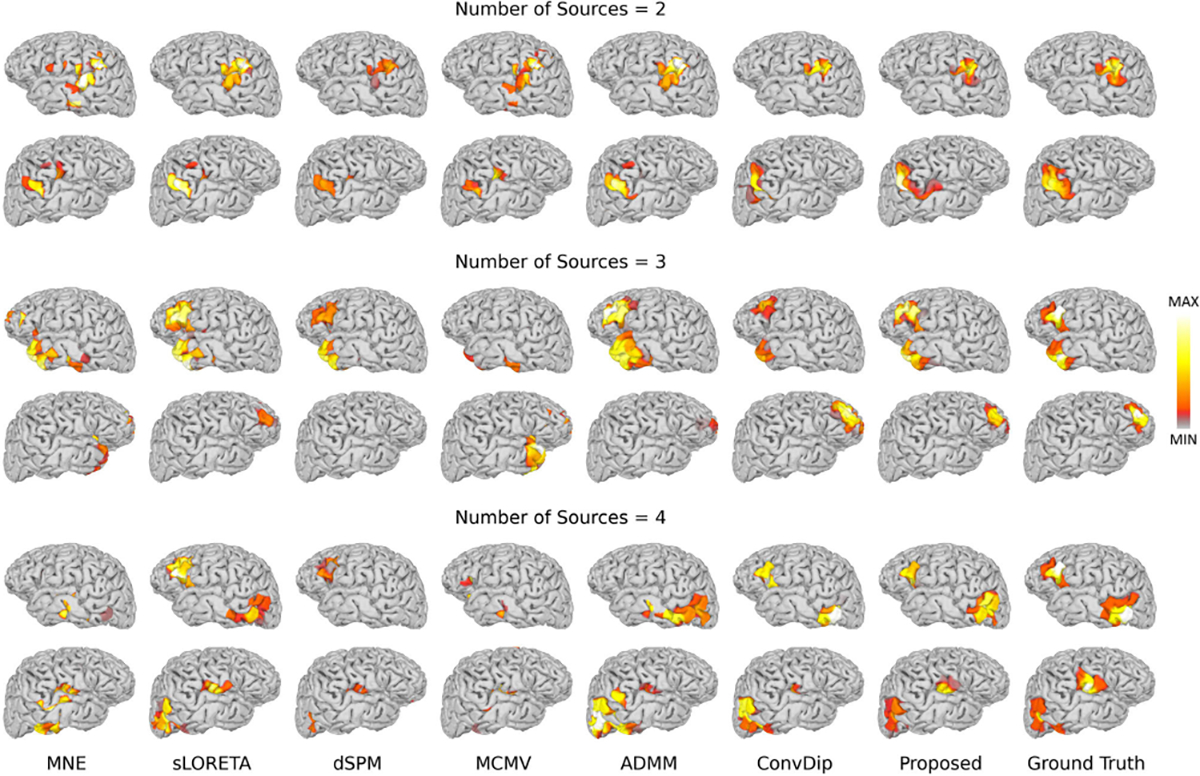
Brain sources reconstruction by different ESI algorithms with SNR = 30 dB and LNs = 3 for multiple sources.

To further assess the performance of MMDF-ANN, we conducted experiments with multiple simultaneously activated sources. The number of sources $\left(N_{s}\right)$ was set to 2, 3, and 4, with MMDF-ANN and benchmark algorithms employed for the reconstruction of multiple sources. The performance comparison on AUPRC is summarized in Table II. The boxplots for different numbers of sources with SNR=30 dB and LNs=3 are provided in Fig. 5. Reconstructed source activities for SNR=30 dB and LNs=3 with different number of sources are shown in Fig. 6.

From the results, we can see that: with the increase in the number of sources, there is a reduction in AUPRC for all algorithms, and the superiority of MMDF-ANN is becoming less pronounced. This indicates the complexity and difficulty inherent in accurately reconstructing multiple sources. However, the performance of deep learning models significantly depends on the diversity of the training set.

### B. Hyperparameter Tuning With Bayesian Optimization

The loss function for the proposed MMDF-ANN contains two adjustable hyperparameters: the weight α and the kernel width ρ controlling the penalty degree for source solutions with different geodesic distances. To achieve optimal source reconstruction performance with MMDF-ANN, we employed Bayesian optimization [65] to determine the optimal values of α and ρ within the specified range of 0.001 to 1. The training set and the validation set were split in a ratio of 70% and 30%. The initial values of α and ρ were randomly selected. The number of trials was 50, with the objective value for each trial determined by the mean AUPRC of results on the validation set.

**Fig. 7.**
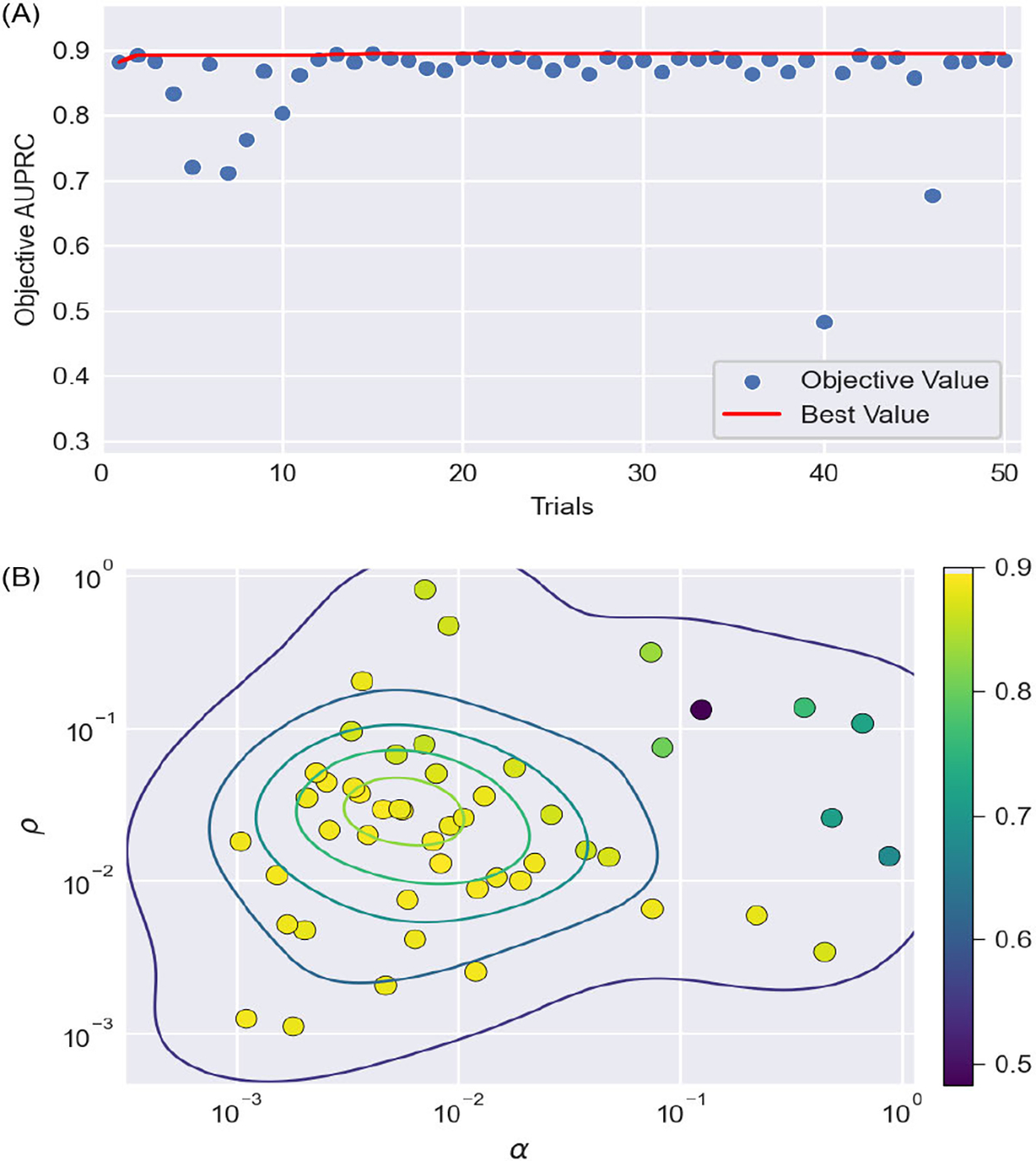
Hyperparameter tuning using Bayesian optimization with AUPRC as objective. There are two subplots: (A) optimization history, (B) selected hyperparameters and the obtained objective value from each trail.

The optimization history and corresponding hyperparameters in each trial were presented in Fig. 7, from which we can see that:

1. The performance of MMDF-ANN is highly sensitive to changes in the weight α and the kernel width ρ. When α and ρ are set greater than 0.1, it is challenging to attain satisfactory model performance. The identical pairing of α≈0.125 and ρ≈0.132 consistently leads to the poorest model performance with the lowest objective value as 0.483.
2. With the decrease of either α or ρ, the range of the other hyperparameter that can yield optimal model performance broadens. When α ranges from 0.001 to 0.01 and ρ ranges from 0.01 to 0.1, there are multiple combinations of α and ρ yield optimal model performance, yet the optimal hyperparameters were achieved in Trail #15 where α≈0.008 and ρ≈0.013 with the highest objective value as 0.894. Specifically, the corresponding AUPRC values for reconstruction results with varying number of sources are $0.944\pm 0.104\;\left(N_{s}=1\right),\;0.896\pm 0.131\;\left(N_{s}=2\right),\;0.845\pm 0.158\;\left(N_{s}=3\right)$, and $0.827\;\pm 0.166\;\left(N_{s}=4\right)$.

Overall, Bayesian optimization offers a highly efficient, fast-converging, and robust adaptive hyperparameter tuning strategy, which is capable of finding the optimal hyperparameter combination in limited iterations, thus effectively saving computing resources.

### C. Ablation Study

**Fig. 8.**
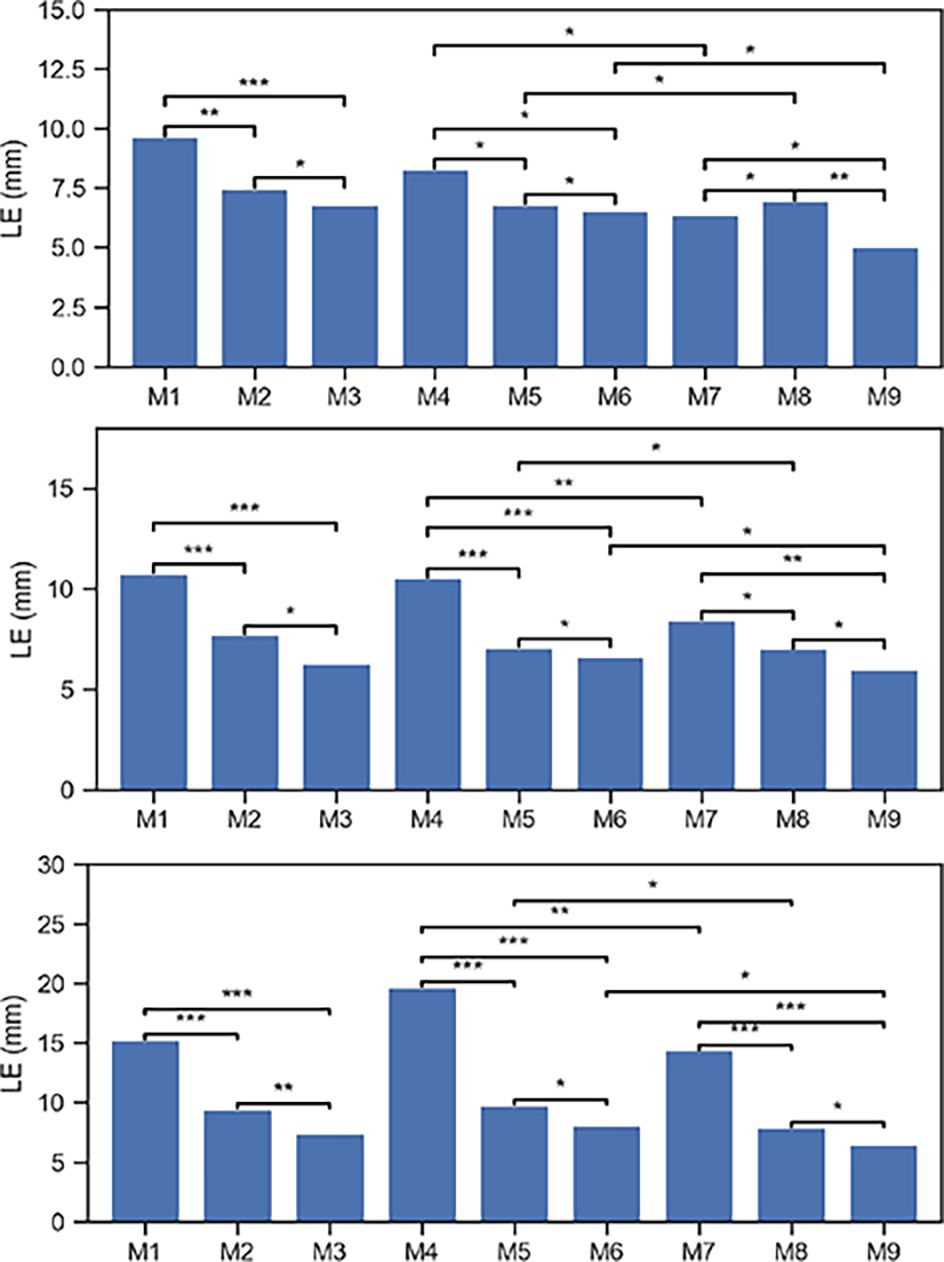
Performance comparison between different models in ablation study for LNs = 3 with SNR = 30 dB (on the top), SNR = 20 dB (in the middle) and SNR = 10 dB (at the bottom). Results are shown as mean values of LE. Asterisks indicate the results of post-hoc paired-sample t-tests: ***-p<0.01, **-p<0.1, *-p<1.

To validate the efficacy of individual components within MMDF-ANN, we conducted a set of ablation experiments. The experimental settings are shown in Table III. The LE comparison for all ablation experiments with LNs=3 and SNR=30 dB, 20 dB, 10 dB are provided in Fig. 8.

It can be concluded from the results that:

1. By the comparison of M3 vs M1-M2, M6 vs M4-M5, and M9 vs M7-M8, we can see that as opposed to the use of single modality EEG or MEG, the combination use of EEG and MEG can significantly reduce the LE level in brain source localization, which suggests that the complementarities between EEG and MEG provide valuable additional information that benefits source estimation.
2. Besides, by the comparison of M1 vs M2, M4 vs M5, and M7 vs M8, we can see models trained on single modality MEG outperform those trained on single modality EEG in most cases. The reason is MEG employs more channels than EEG, and the MEG signal is hardly attenuated by the low conductivity of the skull, resulting in the MEG data contains more comprehensive and accurate information than the EEG data.
3. By the comparison of M1-M3 vs M4-M6, we can see that introducing the dilated convolution can promote the model performance to some extent when SNR is 30 dB. When SNR is set to 10 dB, the LE increases instead.
4. By the comparison of M4 vs M7, M5 vs M8, M6 vs M9, we can see that introducing the topological loss ${ℒ}_{t}$ can effectively improve the accuracy of source localization, especially when only EEG data is adopted. When MEG data is employed, a less obvious reduction in LE is observed. This is because the LE has been reduced to a relatively low level with the incorporation of MEG. It is challenging to further significantly improve model performance by modifying the loss function. This further demonstrates the superior effectiveness and stability of the proposed MMDF-ANN framework.

### D. Experiments on Real Data

To further evaluate the performance of the proposed MMDF-ANN on real data, we applied this framework to both neuroscience studies and clinical applications, which include the source estimation of face perception dataset from SPM (https://www.fil.ion.ucl.ac.uk/spm/data/mmfaces/) and the epilepsy dataset from BrainStorm [60].

#### 1) Evaluation With Face Perception Dataset:

**Fig. 9.**
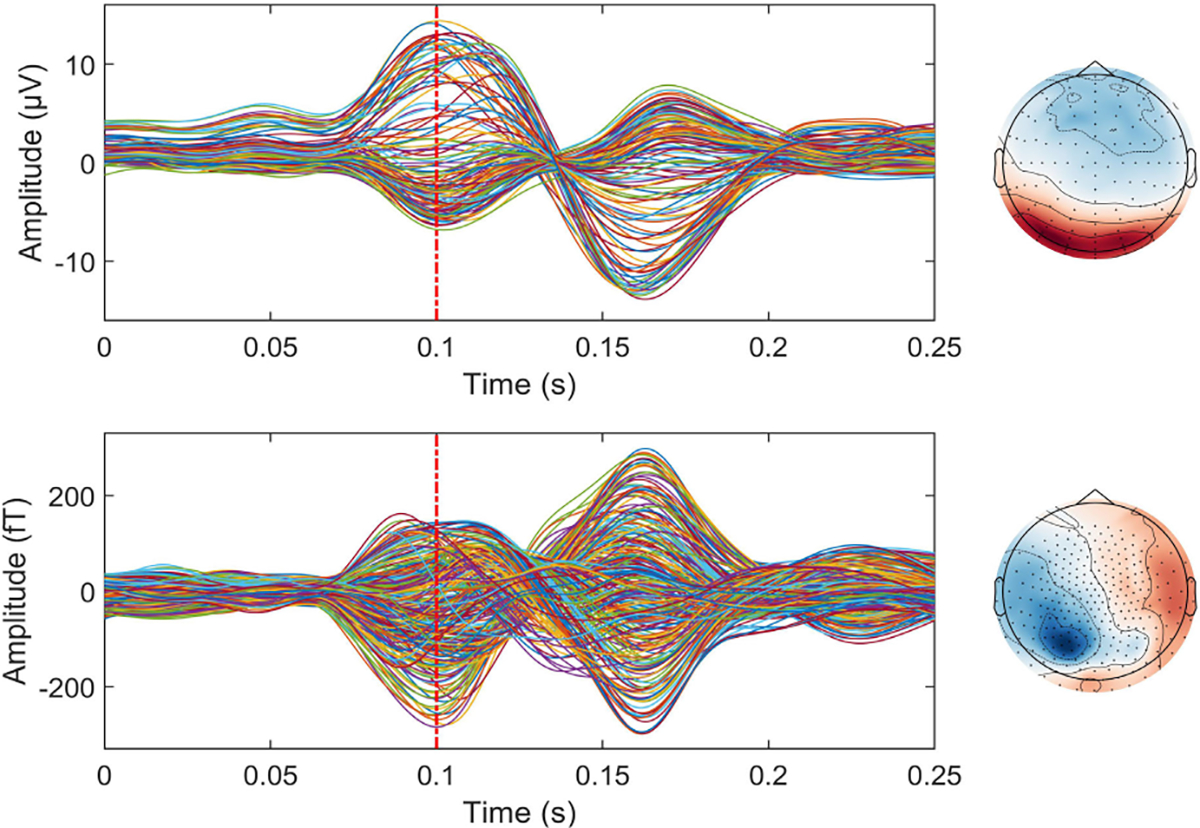
Averaged EEG (on the top) and MEG (at the bottom) time series and corresponding topographic maps of event evoked potentials.

**Fig. 10.**
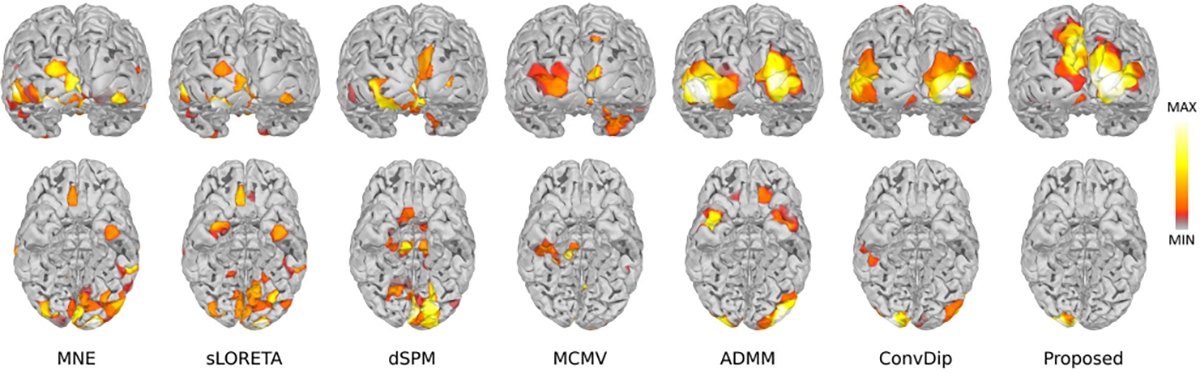
Reconstructed sources for EEG and MEG data from the face perception dataset.

The face perception dataset includes EEG, MEG, and a high resolution anatomical MRI image (aMRI) from a subject who underwent a face perception task. During the task, the subject was asked to make a comparison between *Faces* and *Scrambled* faces. At the same time, a Biosemi system (128 channels) was employed for EEG acquisition, and a CTF system (275 channels) was employed for MEG acquisition. Annotations corresponding to different events are also provided. We conducted head model construction and forward model calculation using MNE-Python toolbox [60], and then we extracted the event-related potentials (ERP) from both EEG and MEG recordings. Finally, we averaged these ERPs (See Fig. 9) and performed brain source localization with MMDF-ANN and benchmark methods (MNE, sLORETA, dSPM, MCMV, ADMM, and ConvDip). The reconstructed source distributions are shown in Fig. 10.

As shown in Fig. 10, the source distributions estimated by MNE, sLORETA, and dSPM produce excessively broad cortical areas that extend beyond the range of the visual area. By contrast, MCMV, ADMM, ConvDip, and the proposed MMDF-ANN provide sparser and more compact and concentrated source reconstructions. Nevertheless, in comparison to MCMV, ADMM and ConvDip, the proposed MMDF-ANN delivers a more precise and cleaner estimation of the visual area.

#### 2) Evaluation With Epilepsy Dataset:

**Fig. 11.**
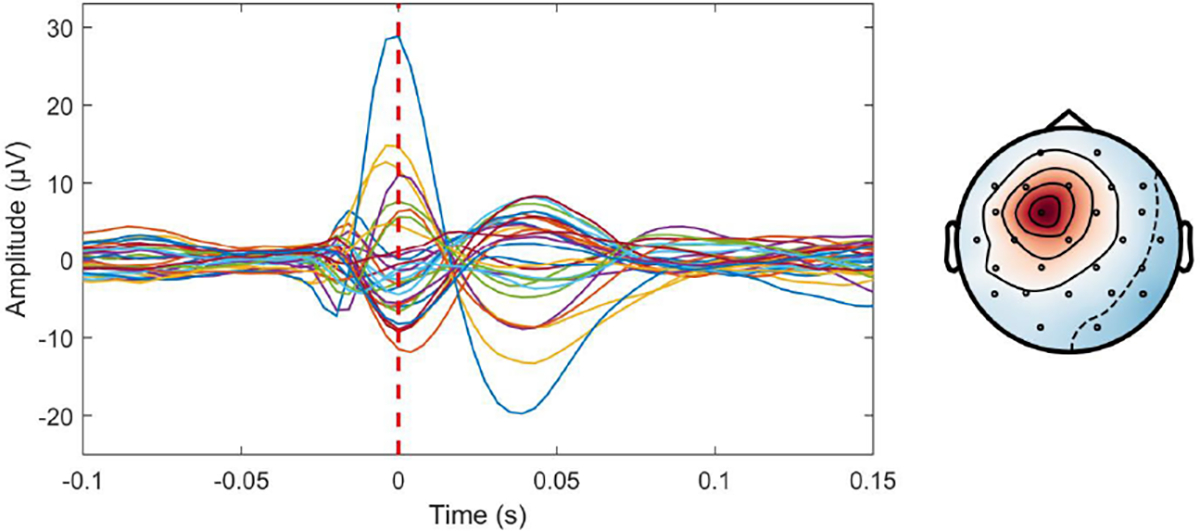
Averaged EEG time series and topographic map of the averaged inter-ictal spike.

**Fig. 12.**
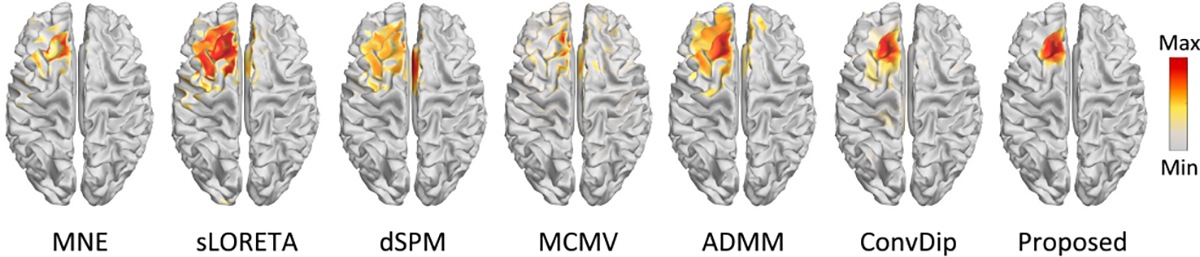
Reconstructed sources for EEG data from the Brainstorm tutorial dataset.

The epilepsy dataset was acquired at the Epilepsy Center Freiburg, Germany, and contains high-resolution 3T epilepsy MRI images and EEG data (29-channel) recorded at 256Hz from a patient with focal epilepsy since the age of 8 years. This patient underwent invasive EEG for epileptogenic area identification, followed by a left frontal tailored resection, and was seizure-free during a 5-year follow-up period. The EEG spikes marked by epileptologists at the Epilepsy Center in Freiburg were also provided. We followed the Brainstorm tutorial to conduct head model construction and forward model calculation, then we averaged the EEG spikes and employed MNE, sLORETA, dSPM, MCMV, ADMM, ConvDip, and MMDF-ANN for epileptic focus localization. Since MEG recordings are not provided, we set EEG as input to both CNN modules in MMDF-ANN. The averaged time series and the topographic map of inter-ictal EEG spikes are plotted in Fig. 11. The reconstructed source distributions of different ESI algorithms are shown in Fig. 12.

It can be seen from Fig. 12 that the reconstructed seizure onset zones (SOZs) by sLORETA, dSPM, MCMV, and ADMM span a wide range of cortical areas and extended beyond the left frontal lobe, whereas MNE, ConvDip, and the proposed MMDF-ANN provide more accurate and concentrated SOZs. Compared to MNE, ConvDip gives a more accurate reconstruction of the extended source area, but there are still observable source activations outside the range of SOZ. By contrast, the proposed MMDF-ANN shows a cleaner reconstruction of the epileptogenic source activation.

## V. Conclusion

In this study, a multi-modal deep fusion framework with attention neural network (MMDF-ANN) is developed to solve the ESI inverse problem by fusing simultaneously recorded EEG and MEG. In the MMDF-ANN, two CNN modules are employed as feature extractors, and the dilated convolution is introduced to expand the receptive field without increasing CNN depth. A channel-wise attention mechanism is designed to selectively learn important feature maps. A topological loss is designed to penalize remote sources with large LE by leveraging the geodesic shortest path defined on the source mesh. To evaluate the performance of the proposed MMDF-ANN, we conducted comprehensive experiments on both simulated and real datasets. The numerical experiments indicate that the performance of MMDF-ANN is superior and more stable compared to the benchmark algorithms, especially when applied to the reconstruction of extended source activations. The results of ablation experiments show that using a multimodal fusion of EEG/MEG can significantly improve the source localization accuracy compared to using single-modality EEG/MEG. The experimental results on real data show that the proposed framework provides a satisfactory reconstruction with a more concentrated and compact source distribution than benchmark algorithms.

## Figures and Tables

**TABLE I:** Performance Comparison Between the Proposed Method and Benchmark Algorithms With Single Source

		Source with LNs=l		Source with LNs=2		Source with LNs=3	

SNR	Method	LE (mm)	AUPRC	LE (mm)	AUPRC	LE (mm)	AUPRC

30dB	MNE	4.991 ± 6.769	0.681 ± 0.207	6.336 ± 7.322	0.557 ± 0.141	8.084 ± 10.508	0.457 ±0.118
	sLORETA	**0.709 ± 2.562**	0.870 ± 0.118	**2.981 ± 4.250**	0.783 ± 0.119	4.890 ± 5.483	0.662 ± 0.121
	dSPM	8.890 ± 11.074	0.678 ± 0.242	12.991 ± 13.672	0.567 ±0.188	23.002 ± 20.596	0.427 ± 0.147
	MCMV	1.688 ± 4.932	0.557 ± 0.202	6.463 ± 11.550	0.425 ± 0.163	10.822 ± 15.528	0.352 ± 0.140
	ADMM	3.254 ± 6.012	**0.895 ± 0.155**	4.416 ± 5.807	0.906 ± 0.083	6.011 ± 5.432	0.869 ± 0.060
	ConvDip	10.828 ± 7.607	0.577 ± 0.259	9.157 ± 6.996	0.812 ± 0.159	9.062 ± 7.171	0.868 ± 0.107
	Proposed	5.046 ± 5.713	0.819 ± 0.181	3.739 ± 6.002	**0.942 ± 0.097**	**4.478 ± 6.072**	**0.965 ± 0.056**

20dB	MNE	5.013 ± 6.824	0.679 ± 0.208	6.218 ± 7.056	0.555 ± 0.140	7.767 ± 10.626	0.457 ±0.117
	sLORETA	**1.447 ± 3.163**	0.858 ± 0.124	**2.967 ± 4.300**	0.778 ± 0.121	5.085 ± 5.787	0.651 ± 0.121
	dSPM	9.569 ± 12.219	0.655 ± 0.238	12.952 ± 13.736	0.554 ± 0.194	26.923 ± 28.094	0.411 ± 0.144
	MCMV	1.688 ± 4.932	0.556 ± 0.203	7.236 ± 12.778	0.426 ± 0.164	11.175 ± 16.327	0.352 ± 0.141
	ADMM	2.793 ± 4.825	**0.895 ± 0.152**	3.798 ± 4.690	0.905 ± 0.081	6.613 ± 5.454	0.864 ± 0.067
	ConvDip	11.343 ± 7.775	0.569 ± 0.251	9.456 ± 6.772	0.813 ± 0.159	9.216 ± 6.820	0.866 ± 0.104
	Proposed	5.978 ± 5.898	0.813 ± 0.185	3.739 ± 6.002	**0.939 ± 0.100**	**4.387 ± 5.513**	**0.964 ± 0.054**

lOdB	MNE	4.919 ± 6.337	0.669 ± 0.203	5.307 ± 5.756	0.550 ± 0.138	7.487 ± 9.334	0.450 ±0.114
	sLORETA	12.543 ± 30.572	0.569 ± 0.236	6.286 ± 6.755	0.629 ± 0.153	9.041 ± 7.622	0.536 ± 0.120
	dSPM	10.190 ± 10.527	0.473 ± 0.221	15.494 ± 15.873	0.442 ±0.185	25.623 ± 20.348	0.337 ± 0.131
	MCMV	**1.688 ± 4.932**	0.557 ± 0.202	7.639 ± 12.645	0.423 ± 0.167	10.065 ± 11.563	0.351 ± 0.142
	ADMM	3.027 ± 5.543	**0.888 ± 0.169**	**4.928 ± 5.744**	0.876 ± 0.092	7.293 ± 6.747	0.801 ± 0.089
	ConvDip	11.616 ± 7.704	0.558 ± 0.262	9.470 ± 7.458	0.786 ± 0.170	9.922 ± 7.279	0.851 ± 0.111
	Proposed	5.608 ± 5.158	0.804 ± 0.181	4.999 ± 6.068	**0.933 ± 0.099**	**5.068 ± 5.543**	**0.943 ± 0.058**

Results are shown as mean ± std.

**TABLE II:** Performance Comparison Between the Proposed Method and Benchmark Algorithms With Multiple Sources

		Source with LNs=l			Source with LNs=2			Source with LNs=3		

*N*_*s*_	Method	SNR=30	SNR=20	SNR=10	SNR=30	SNR=20	SNR=10	SNR=30	SNR=20	SNR=10

2	MNE	0.544 ± 0.135	0.546 ± 0.133	0.538 ± 0.146	0.424 ±0.121	0.424 ±0.118	0.410 ± 0.117	0.327 ± 0.116	0.325 ± 0.116	0.322 ±0.117
	sLORETA	**0.846 ± 0.075**	**0.807 ± 0.113**	0.490 ± 0.215	0.753 ± 0.065	0.740 ± 0.063	0.559 ± 0.133	0.668 ± 0.073	0.657 ± 0.076	0.531 ± 0.091
	dSPM	0.631 ± 0.192	0.599 ± 0.193	0.337 ± 0.206	0.493 ± 0.121	0.479 ± 0.123	0.342 ± 0.143	0.393 ± 0.108	0.378 ± 0.106	0.314 ± 0.125
	MCMV	0.326 ± 0.154	0.327 ± 0.157	0.318 ± 0.156	0.265 ± 0.099	0.264 ± 0.101	0.259 ± 0.096	0.213 ± 0.081	0.213 ± 0.081	0.215 ± 0.079
	ADMM	0.814 ± 0.104	0.805 ± 0.110	**0.745 ± 0.183**	0.868 ± 0.064	0.863 ± 0.070	0.744 ± 0.145	0.832 ± 0.080	0.808 ± 0.091	0.696 ± 0.137
	ConvDip	0.538 ± 0.171	0.542 ± 0.184	0.520 ± 0.165	0.785 ± 0.103	0.783 ± 0.104	0.730 ± 0.122	0.884 ± 0.070	0.883 ± 0.067	0.841 ± 0.100
	Proposed	0.696 ± 0.129	0.680 ± 0.145	0.618 ± 0.155	**0.908 ± 0.060**	**0.901 ± 0.066**	**0.839 ± 0.110**	**0.942 ± 0.040**	**0.941 ± 0.039**	**0.892 ± 0.071**

3	MNE	0.394 ± 0.120	0.392 ± 0.121	0.383 ± 0.126	0.294 ± 0.085	0.294 ± 0.084	0.286 ± 0.088	0.222 ± 0.071	0.222 ± 0.072	0.216 ± 0.069
	sLORETA	**0.776 ± 0.088**	**0.746 ± 0.106**	0.471 ±0.177	0.695 ± 0.072	0.683 ± 0.073	0.535 ± 0.112	0.591 ± 0.081	0.581 ± 0.084	0.478 ± 0.078
	dSPM	0.516 ± 0.165	0.498 ± 0.159	0.301 ± 0.154	0.405 ± 0.110	0.394 ± 0.109	0.307 ±0.112	0.320 ± 0.096	0.314 ± 0.098	0.257 ± 0.070
	MCMV	0.180 ± 0.102	0.178 ± 0.102	0.178 ± 0.099	0.167 ± 0.077	0.167 ± 0.078	0.166 ± 0.077	0.153 ± 0.074	0.152 ± 0.073	0.154 ± 0.070
	ADMM	0.738 ± 0.094	0.732 ± 0.096	**0.654 ± 0.145**	**0.822 ± 0.067**	**0.809 ± 0.074**	0.683 ±0.117	0.782 ± 0.070	0.757 ± 0.085	0.586 ± 0.151
	ConvDip	0.425 ± 0.147	0.419 ± 0.149	0.407 ± 0.153	0.708 ± 0.124	0.701 ± 0.123	0.623 ± 0.125	0.817 ± 0.076	0.808 ± 0.079	0.744 ± 0.094
	Proposed	0.543 ± 0.151	0.531 ± 0.148	0.483 ± 0.148	0.808 ± 0.106	0.792 ± 0.110	**0.695 ± 0.149**	**0.859 ± 0.067**	**0.843 ± 0.078**	**0.761 ± 0.112**

4	MNE	0.318 ± 0.103	0.317 ± 0.105	0.302 ± 0.104	0.214 ± 0.073	0.212 ± 0.074	0.208 ± 0.074	0.182 ± 0.060	0.181 ± 0.059	0.178 ± 0.060
	sLORETA	**0.740 ± 0.094**	**0.689 ± 0.094**	0.394 ± 0.154	0.632 ± 0.076	0.619 ± 0.078	0.471 ±0.113	0.533 ± 0.071	0.521 ± 0.072	0.432 ± 0.086
	dSPM	0.427 ± 0.136	0.390 ± 0.135	0.243 ±0.114	0.336 ± 0.084	0.328 ± 0.081	0.272 ± 0.091	0.286 ± 0.072	0.281 ± 0.073	0.240 ± 0.077
	MCMV	0.117 ± 0.095	0.117 ± 0.096	0.118 ± 0.093	0.136 ± 0.079	0.136 ± 0.080	0.136 ± 0.077	0.146 ± 0.058	0.146 ± 0.058	0.146 ± 0.060
	ADMM	0.693 ± 0.100	0.677 ± 0.103	**0.587 ± 0.149**	**0.764 ± 0.090**	**0.742 ± 0.096**	0.556 ± 0.155	0.716 ± 0.109	0.679 ± 0.113	0.492 ± 0.155
	ConvDip	0.385 ± 0.114	0.380 ± 0.109	0.359 ± 0.110	0.647 ± 0.104	0.635 ± 0.109	0.585 ± 0.134	0.770 ± 0.088	0.763 ± 0.093	0.669 ± 0.118
	Proposed	0.426 ± 0.147	0.418 ± 0.149	0.358 ± 0.142	0.689 ± 0.120	0.678 ± 0.115	**0.595 ± 0.140**	0.769 ± 0.093	**0.764 ± 0.097**	**0.691 ± 0.117**

Results are shown as mean ± std of AUPRC.

**TABLE III:** Design of Ablation Experiments

Model	EEG	MEG	Dilation Rate	${ℒ}_{t}$

M1	✓		1	
M2		✓	1	
M3	✓	✓	1	
M4	✓		2	
M5		✓	2	
M6	✓	✓	2	
M7	✓		2	✓
M8		✓	2	✓
M9	✓	✓	2	✓
